# Association of Parental Socioeconomic Status and Newborn Telomere Length

**DOI:** 10.1001/jamanetworkopen.2020.4057

**Published:** 2020-05-04

**Authors:** Dries S. Martens, Bram G. Janssen, Esmée M. Bijnens, Diana B. P. Clemente, Paolo Vineis, Michelle Plusquin, Tim S. Nawrot

**Affiliations:** 1 Centre for Environmental Sciences, Hasselt University, Hasselt, Belgium; 2 Medical Research Council–Health Policy Agency, Centre for Environment and Health, Department of Epidemiology and Biostatistics, Imperial College London, London, United Kingdom; 3 Department of Public Health and Primary Care, Leuven University, Leuven, Belgium

## Abstract

**Importance:**

Low socioeconomic status is associated with higher all-cause mortality and risks for aging-related diseases. Biological aging is a potential process underlying health conditions related to social disadvantages, which may be present from birth onward.

**Objective:**

To evaluate the association of parental socioeconomic status with telomere length (TL) at birth, a marker of biological aging.

**Design, Setting, and Participants:**

This prospective birth cohort study was conducted among 1504 mother-newborn pairs in Belgium recruited between February 1, 2010, and July 1, 2017.

**Exposures:**

Parental socioeconomic measures, including maternal educational level, occupation, paternal educational level, and neighborhood income based on median annual household income.

**Main Outcomes and Measures:**

Mean relative TL was measured in cord blood and placental tissue. By constructing a principal component, an integrative socioeconomic measure was derived that integrates parental socioeconomic status and neighborhood income. Multivariable adjusted regression analyses were performed to associate the integrative socioeconomic measure and TL at birth.

**Results:**

In 1026 newborns (517 boys; mean [SD] gestational age, 39.2 [1.4] weeks), a higher socioeconomic status was associated with longer cord blood TL and placental TL. Each unit increment in the integrative socioeconomic status measure was associated with 2.1% (95% CI, 0.9%-3.4%; *P* < .001) longer cord blood TL in boys, while no association was observed for girls (0.5% longer cord blood TL; 95% CI, −0.9% to 1.8%; *P* = .50). The sex-specific socioeconomic status interaction revealed a stronger association in boys compared with newborn girls (1.6%; 95% CI, 0.02%-3.3%; *P* = .047 for interaction). In placental tissue, higher socioeconomic status was associated with 1.8% (95% CI, 0.3%-3.3%; *P* = .02) longer TL in newborn boys but not in girls (0.4% longer TL; 95% CI, −1.2% to 2.0%; *P* = .63). For placental tissue, no sex and socioeconomic status interaction on TL was observed (1.4%; 95% CI, −0.5% to 3.4%; *P* = .16 for interaction).

**Conclusions and Relevance:**

This study suggests that parental socioeconomic status is associated with newborn TL, especially in boys. The results indicate that familial social economic factors are associated with the potential cellular longevity of the next generation, with a potential higher transgenerational vulnerability for newborn boys.

## Key Points

**Question:**

Is parental socioeconomic status associated with telomere length at birth?

**Findings:**

This cohort study found that early biological aging, reflected by shorter telomere length in newborns, was associated with lower parental socioeconomic status. A higher vulnerability was observed in boys compared with girls.

**Meaning:**

Addressing socioeconomic disadvantages very early in life may improve molecular longevity and overall health for the next generation.

## Teaser

This cohort study evaluates the association of parental socioeconomic status with telomere length at birth, a marker of biological aging.

## Introduction

Low socioeconomic status (SES) is associated with a higher prevalence of risk factors,^1^ increased risk in developing age-related diseases, overall mortality,^2^ and a reduced life expectancy of up to 2.1 years.^3^ A large variability in socioeconomic inequalities exists between countries; however, associations between SES and mortality are consistent between countries, but with differences in the magnitude of effects.^4^ This finding suggests that SES differences in association with health, disease, and mortality are observed universally, even in affluent countries, which may be partly explained by differences in health behaviors.^4,5^

Telomere length (TL) is a biological marker of aging. After each cellular division, telomeres shorten; increased oxidative stress and inflammation may accelerate telomere shortening. At birth, TL is highly variable, may be associated with later-life TL, and may be a determinant of an individual’s natural lifespan.^6,7,8^ Short TL later in life has been associated with increased disease risk and mortality, and may have an origin very early in life, as reflected by TL at birth.^9^ Heritable, environmental, and lifestyle factors are associated with an individual’s TL. Studying determinants of TL at birth may lead to a better understanding of the initial TL setting and are essential in understanding their potential role in the developmental origins of health and disease. Maternal perturbations during pregnancy have been associated with newborn TL, including prepregnancy body mass index (BMI),^10^ air pollution,^11^ residential proximity to major roads,^12^ stress,^13^ and smoking.^14^

It has been suggested that accelerated cellular or biological aging plays a role in the link between SES and health.^15^ Meta-analytical evidence suggests an association between SES and adult TL.^16^ Social disadvantages in early life may induce persistent biological changes.^17^ In this regard, recent studies, using a limited number of participants, showed associations between newborn TL and maternal educational levels, with potential sex-specific associations.^18,19^ However, other large studies of newborns were not able to confirm these associations.^9,20,21^

We hypothesize that parental SES may set the TL of the next generation. We tested our hypothesis in newborns from the ENVIRONAGE (Environmental Influence on Aging in Early Life) birth cohort,^22^ and in addition, we evaluated whether these associations were different by newborn sex.

## Methods

### Study Population and Data Collection

General study procedures for the ongoing ENVIRONAGE birth cohort have been described previously.^22^ For this study, a total of 1504 mother-newborn pairs were recruited from February 1, 2010, to July 1, 2017. The selection criteria included the mother’s ability to fill out questionnaires in Dutch. We achieved an overall participation rate of 61% (1504 of 2465). Selection of eligible mother-newborn pairs is shown in eFigure 1 in the Supplement. Detailed descriptions of demographic and perinatal variables obtained via questionnaire and medical records are provided in eMethods 1 in the Supplement. The study protocol was approved by the Ethical Committee of Hasselt University and East-Limburg Hospital in Genk and has been carried out according to the Declaration of Helsinki.^69^ This study was performed according to the Strengthening the Reporting of Observational Studies in Epidemiology (STROBE) reporting guideline. Written informed consent was obtained from all participants.

### Parental SES and Neighborhood Income

Educational attainment was assessed as the highest educational level successfully completed using the International Standard Classification of Education.^23^ Maternal and paternal educational level was coded as low, middle, and high (eMethods 2 in the Supplement). Maternal occupational levels (low, middle, and high) were assessed using the Standard Occupational Classification (eMethods 2 in the Supplement).^24^ We chose not to ask about income because, based on experience in other population-based studies in Belgium,^25,26^ this question has been considered as a violation of privacy. We assessed neighborhood income, based on median annual household income (eMethods 2 in the Supplement), as this might reflect contextual associations and the geographical dispersion of potential risk factors.^27^

### Relative TL Measurement

Details on cord blood, placenta collection, cord blood cell differential analyses and TL measurement using quantitative polymerase chain reaction are described in eMethods 3 in the Supplement.^10,22^ Telomere length was expressed as the ratio of telomere copy number to single-copy gene number (T/S) relative to the mean T/S ratio of the entire sample set within each measured batch. The reliability of our assay was assessed by calculating the interclass correlation coefficient of triplicate measures (T/S ratios, telomere copy number and single-copy gene number measures) (eMethods 3 in the Supplement).

### Statistical Analysis

All analyses were performed using SAS, version 9.4 (SAS Institute Inc). All *P* values were from 2-sided tests and results were deemed statistically significant at *P* < .05. Normality was tested using the Shapiro-Wilk test. Telomere lengths were log_10_-transformed to improve normality. We assessed the distributions of continuous variables (analysis of variance) and proportions of categorical variables (χ^2^ test) across the different classes of maternal educational levels. An integrative SES variable was constructed by a principal component that combines the different SES measures (maternal educational level, occupation, paternal educational level, and neighborhood income) using the PROC PRINQUAL procedure in SAS. We used multiple linear regression models to associate the integrative SES variable with cord blood and placental TL. First, we constructed a directed acyclic graph using a priori selected covariates including determinants of newborn and adult TL and variables associated with SES, including parental ages, prepregnancy BMI, maternal smoking, parity, pregnancy complications, cesarean delivery, newborn sex, gestational age, birth weight, and newborn race/ethnicity. Based on the directed acyclic graph, a minimal adjustment model was constructed including maternal age, paternal age, and newborn race/ethnicity, which is required to observe the total association of SES with TL. Second, a full adjustment model was applied, including all aforementioned covariates. All models were adjusted for the variable “batch” to account for the measurement of TL in 2 separate batches. Telomere length at birth differs between boys and girls,^11^ and the association between SES and TL may be sex-specific.^18^ Therefore, we formally tested effect modification of newborn sex by adding an interaction term between the integrative SES measure and newborn sex. Newborn sex-specific estimates are reported from these interaction models.

Several potential mediators (see eMethods 4 in the Supplement for selection) of the association between SES (exposure variable) and newborn TL (outcome variable) were evaluated. This was accomplished by decomposing the total effect into a direct effect (ie, exposure effect on outcome at a fixed level of the mediator) and an indirect effect (ie, exposure effect on the outcome that operates through the mediator).

In sensitivity analyses, we adjusted our models for long-term residential exposure to particulate matter with an aerodynamic diameter of 2.5 μm or smaller (eMethods 5 in the Supplement), maternal fruit and vegetable consumption, maternal physical activity, and blood cell differentials (cord blood models) or excluded newborns of African descent, mothers with pregnancy complications or cesarean delivery, and smokers.

As a secondary analysis, and to evaluate the associations with individual SES indicators, we performed multivariable adjusted models to associate maternal educational level, occupation, paternal educational level, and neighborhood income with newborn TL. Sex-specific estimates are reported from models including a newborn sex × SES interaction term.

## Results

### Study Population Characteristics

Newborn, maternal, and paternal demographic characteristics by maternal educational classes are provided in Table 1. Mothers had a mean (SD) age of 29.5 (4.6) years, and fathers had a mean (SD) age of 32.0 (5.3) years. Most mothers (652 of 1258 [51.8%]) had a university or college degree, 467 of 1258 (37.1%) obtained a secondary school degree, and 139 of 1258 (11.0%) did not obtain any diploma. Mothers in the lowest educational category included more smokers than those in the middle and highest educational category (51 of 139 [36.7%] vs 74 of 467 [15.8%] vs 25 of 652 [3.8%]), had more children (≥3 children: 32 of 139 [23.0%] vs 69 of 467 [14.8%] vs 51 of 652 [7.8%]), had a higher mean (SD) prepregnancy BMI (25.0 [5.4] vs 25.0 [4.9] vs 24.2 [4.3]; calculated as weight in kilograms divided by height in meters squared), consumed less fruit and vegetables (≥3 portions per day: 21 of 132 [15.9%] vs 72 of 437 [16.5%] vs 154 of 632 [24.4%]), were less physically active (low physical activity: 48 of 133 [36.1%] vs 150 of 435 [34.5%] vs 177 of 633 [28.0%]), and were younger (mean [SD] age: 28.3 [5.9] vs 28.6 [4.8] vs 30.4 [3.8] years). A total of 190 mothers had no job during pregnancy, includind 24 students and 166 mothers who could be classified into job categories based on their job status prior to pregnancy. The newborns (517 boys) had a mean (SD) gestational age of 39.2 (1.4) weeks and a mean birth weight of 3421 (471) g. A total of 1107 of 1258 newborns (88.0%) were of European descent. Maternal occupation was correlated with maternal educational level (*r* = 0.71; *P* < .001), maternal educational level was correlated with paternal educational level (*r* = 0.46; *P* < .001), and neighborhood income was correlated with maternal educational level (*r* = 0.23; *P* < .001), maternal occupation (*r* = 0.25; *P* < .001), and paternal occupation (*r* = 0.21; *P* < .001) (eFigure 2 in the Supplement). Characteristics of the integrative SES measure are provided in the eTable in the Supplement. Maternal educational level was strongly correlated with the integrative SES measure (*r* = 0.87; *P* < .001), as was maternal occupation (*r* = 0.85; *P* < .001) (eFigure 2 in the Supplement). Cord blood TL ranged from 0.49 to 1.75 and placental TL ranged from 0.38 to 2.0 and were correlated (*r* = 0.42; *P* < .001). Girls had 5.2% (95% CI, 3.1%-7.2%; *P* < .001) longer cord blood telomeres and 5.1% (95% CI, 2.6%-7.6%; *P* < .001) longer placental telomeres compared with boys.

**Table 1.** Population Characteristics of 1258 Mother-Newborn Pairs According to Maternal Educational Classes

Characteristic	Maternal educational classes, No. (%)			*P* value for trend
	Low (n = 139)	Middle (n = 467)	High (n = 652)	
**Newborn**				
Girls	56 (40.3)	221 (47.3)	335 (51.4)	.046
Ethnicity, European grandparents				
0	35 (25.2)	71 (15.2)	37 (5.7)	<.001
1	3 (2.2)	4 (0.9)	1 (0.1)	
2	8 (5.7)	29 (6.2)	22 (3.4)	
3	3 (2.2)	12 (2.6)	11 (1.7)	
4	90 (64.7)	351 (75.1)	581 (89.1)	
Gestational age, mean (SD), wk	39.1 (1.4)	39.3 (1.3)	39.2 (1.5)	.33
Birth weight, mean (SD), g	3326 (411)	3410 (479)	3451 (474)	.01
Telomere length, geometric mean (IQR), T/S ratio				
Cord blood	0.97 (0.85-1.12)	0.98 (0.87-1.13)	1.01 (0.89-1.16)	.007
Placental	0.98 (0.82-1.11)	0.99 (0.84-1.17)	1.02 (0.87-1.20)	.048
**Maternal**				
Age, mean (SD), y	28.3 (5.9)	28.6 (4.8)	30.4 (3.8)	<.001
BMI, mean (SD)	25.0 (5.4)	25.0 (4.9)	24.2 (4.3)	.01
Smoking				
Never smoker	62 (44.6)	263 (56.3)	490 (75.2)	<.001
Stopped smoker	26 (18.7)	130 (27.8)	137 (21.0)	
Smoker	51 (36.7)	74 (15.8)	25 (3.8)	
Parity				
1	53 (38.1)	237 (50.7)	368 (56.5)	<.001
2	54 (38.9)	161 (34.5)	233 (35.7)	
≥3	32 (23.0)	69 (14.8)	51 (7.8)	
Job ranking, No./total No. (%)^a^				
Low	114/129 (88.4)	188/419 (44.9)	26/638 (4.1)	<.001
Middle	12/129 (9.3)	199/419 (47.5)	107/638 (16.8)	
High	3/129 (2.3)	32/419 (7.6)	505/638 (79.1)	
Fruit, vegetables consumption, No./total No. (%)^b^				
<1 portion/d	34/132 (25.8)	57/437 (13.0)	50/632 (7.9)	<.001
1 portion/d	39/132 (29.5)	157/437 (35.9)	180/632 (28.5)	
2 portions/d	38/132 (28.8)	151/437 (34.6)	248/632 (39.2)	
≥3 portions/d	21/132 (15.9)	72/437 (16.5)	154/632 (24.4)	
Physical activity, No./total No. (%)^b^				
Low	48/133 (36.1)	150/435 (34.5)	177/633 (28.0)	.02
Middle	21/133 (15.8)	80/435 (18.4)	157/633 (24.8)	
High	64/133 (48.1)	205/435 (47.1)	299/633 (47.2)	
Pregnancy complication	12 (8.6)	72 (15.4)	85 (13.0)	.11
Cesarean delivery	6 (4.3)	17 (3.6)	27 (4.1)	.89
Entire pregnancy PM2.5, mean (SD), μg/m^3^^c^	12.8 (2.1)	12.6 (2.5)	12.6 (2.6)	.67
Median annual income, mean (SD), €^d^	23 562 (3375)	24 284 (3363)	25 637 (3204)	<.001
**Paternal**				
Educational level, No./total No. (%)^e^				
Low	54/105 (51.4)	57/373 (15.3)	36/587 (6.1)	<.001
Middle	46/105 (43.8)	245/373 (65.7)	226/587 (38.5)	
High	5/105 (4.8)	71/373 (19.0)	325/587 (55.4)	
Age, mean (SD), y	30.8 (6.6)	31.2 (5.6)	32.7 (4.7)	<.001

Abbreviations: BMI, body mass index (calculated as weight in kilograms divided by height in meters squared); IQR, interquartile range; PM2.5, particulate matter with an aerodynamic diameter 2.5 μm or smaller; T/S, telomere copy number to single-copy gene number.

^a^ Data available for 1186 mother-newborn pairs.

^b^ Data available for 1201 mother-newborn pairs.

^c^ Data available for 1103 mother-newborn pairs.

^d^ Data available for 1244 mother-newborn pairs.

^e^ Data available for 1065 mother-newborn pairs.

### Newborn TL and Integrative SES

In unadjusted (Figure) and adjusted models, SES was positively associated with cord blood and placental TLs (Table 2). Effect estimates were stronger in boys compared with girls, and interaction terms of the adjusted models suggest a sex-specific association between SES and cord blood TL (1.6%; 95% CI, 0.02%-3.3%; *P* = .047 for interaction). For placenta, no sex and SES interaction on TL was observed (1.4%; 95% CI, −0.5% to 3.4%; *P* = .16 for interaction). Each unit increment in the integrative SES measure was associated with 1.4% (95% CI, 0.4%-2.4%; *P* = .006) longer cord blood TL and 1.2% (95% CI, 0.0%-2.3%; *P* = .048) longer placental TL. For newborn boys, each unit increment was associated with 2.1% (95% CI, 0.9%-3.4%; *P* < .001) longer cord blood TL and 1.8% (95% CI, 0.3%-3.3%; *P* = .02) longer placental TL. For newborn girls, each unit increment was associated with a statistically insignificant 0.5% (95% CI, −0.9% to 1.8%; *P* = .50) longer cord blood TL and a statistically insignificant 0.4% (95% CI, −1.2% to 2.0%, *P* = .63) longer placental TL.

**Figure.**
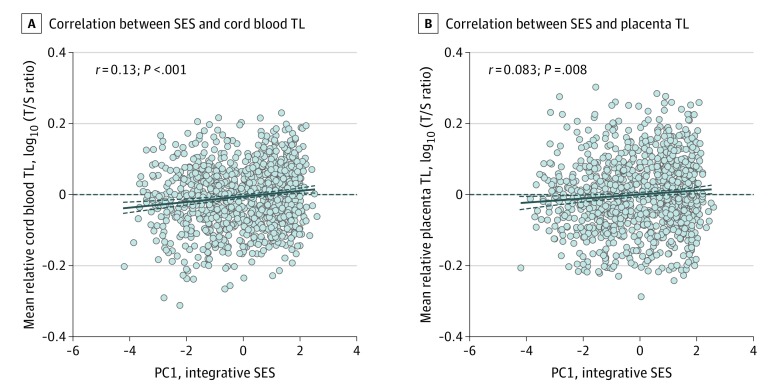
Scatterplot Depicting the Association Between Integrative Socioeconomic Status (SES) Measure and Newborn Telomere Length (TL) A, Unadjusted Pearson correlation between SES and mean relative cord blood TL (n = 1026). B, Unadjusted Pearson correlation between SES and average relative placenta TL (n = 1026). PC1 indicates principal component 1 as a reflector of the integrative SES measure; T/S, telomere copy number to single-copy gene number. The thick dark blue solid line is the regression line, and the dark blue dashed lines above and below it indicate the 95% CI.

**Table 2.** Association Between Integrative SES Measure and Newborn Telomere Length^a^

Model^b^	Total population (n = 1026)		Boys (n = 517)		Girls (n = 509)		*P* value for interaction^c^
	% Difference (95% CI)	*P* value	% Difference (95% CI)	*P* value	% Difference (95% CI)	*P* value	
**Cord blood**							
Model A	1.8 (1.0 to 2.6)	<.001	2.4 (1.3 to 3.5)	<.001	1.0 (−0.2 to 2.2)	.09	.10
Model B	1.4 (0.5 to 2.3)	.003	2.0 (0.9 to 3.2)	<.001	0.5 (−0.7 to 1.8)	.51	.07
Model C	1.4 (0.4 to 2.4)	.006	2.1 (0.9 to 3.4)	<.001	0.5 (−0.9 to 1.8)	.50	.047
**Placenta**							
Model A	1.3 (0.3 to 2.3)	.008	1.8 (0.5 to 3.1)	.008	0.7 (−0.7 to 2.2)	.32	.29
Model B	1.0 (−0.03 to 2.1)	.06	1.5 (0.2 to 2.9)	.03	0.3 (−1.2 to 1.8)	.67	.22
Model C	1.2 (0.0 to 2.3)	.048	1.8 (0.3 to 3.3)	.02	0.4 (−1.2 to 2.0)	.63	.16

Abbreviations: PC1, principal component 1; SES, socioeconomic status.

^a^ Estimates provided as a % difference (95% CI) in telomere length for 1-unit increment in PC1, integrative SES.

^b^ Model A: adjusted for telomere batch. Model B: minimal adjusted model for maternal age, paternal age, and newborn race/ethnicity, based on covariate selection using directed acyclic graphs, additionally adjusted for telomere batch. Model C: full adjusted model for maternal age, paternal age, maternal prepregnancy body mass index, maternal smoking, parity, pregnancy complications, cesarean delivery, newborn sex, gestational age, birth weight, newborn race/ethnicity, and telomere batch.

^c^ Represents the *P* value for the interaction term newborn sex × PC1.

### Mediation Analysis

Prepregnancy BMI partially mediated the association between the integrative SES measure and cord blood TL in the total population. The estimated proportion of mediation was 10.9% (95% CI, 4.6%-12.4%; *P* < .001), with an indirect effect of 0.6% (95% CI, 0.1%-1.2%; *P* = .02). No mediation was observed for placental TL. Other potential mediators including maternal smoking, exposure to particulate matter with an aerodynamic diameter of 2.5 μm or smaller, birth weight, maternal fruit and vegetable consumption, and maternal physical activity did not fulfill the assumption of a mediator being associated with both exposure and outcome.

### Sensitivity Analyses

Our findings were robust when excluding newborns of African descent or mothers experiencing pregnancy complications or who underwent a cesarean delivery. Adjustment for maternal fruit and vegetable consumption or physical activity did not alter our results in the total population (eFigure 3 in the Supplement) and for boys and girls separately (eFigure 4 in the Supplement). Associations in cord blood were robust for adjustment for blood cell differentials. Adjusting for residential exposure to particulate matter with an aerodynamic diameter of 2.5 μm or smaller and excluding smokers attenuated the estimates observed in placental tissue for the total population and for boys.

### Newborn TL and Individual Indicators of SES

Maternal occupation was associated with cord blood TL in the total population (compared with high-level maternal occupation, difference in TL for middle-level maternal occupation, –4.3% [95% CI, –6.9% to –1.6%]; and difference in TL for low-level maternal occupation, –4.0% [95% CI, –6.9% to –1.1%]) (Table 3). In boys, maternal educational level (compared with high-level maternal educational level, difference in TL for middle-level maternal educational level, –4.0% [95% CI, –7.2% to –0.7%]; and difference in TL for low-level maternal educational level, –6.7% [95% CI, –11.3% to –1.9%]), occupation (compared with high-level maternal occupation, difference in TL for middle-level maternal occupation, –4.6% [95% CI, –8.3% to –0.9%]; and difference in TL for low-level maternal occupation, –7.3% [95% CI, –10.9% to –3.4%]), and paternal educational level (compared with high-level paternal educational level, difference in TL for middle-level paternal educational level, –0.5% [95% CI, –4.0% to 3.1%]; and difference in TL for low-level paternal educational level, –6.3% [95% CI, –11.1% to –1.1%]) were associated with cord blood TL. We observed an interaction for newborn sex and maternal education, occupation, and paternal education.

**Table 3.** Individual SES Indicators and Cord Blood TL^a^

Indicator	No.	Total population		No.	Boys			No.		Girls			*P* value for interaction^b^	
		% Difference (95% CI)	*P* value		% Difference (95% CI)	*P* value			% Difference (95% CI)		*P* value		
Maternal educational level^c^	1258			646			612						
High	652	1 [Reference]	.15	317	1 [Reference]	.008	335		1 [Reference]		.88	.039	
Middle	467	−2.0 (−4.4 to 0.5)		246	−4.0 (−7.2 to −0.7)		221		0.0 (−3.3 to 3.5)				
Low	139	−3.3 (−7.0 to 0.7)		83	−6.7 (−11.3 to −1.9)		56		1.4 (−4.3 to 7.5)				
Maternal occupation^c^	1186			601			585						
High	540	1 [Reference]	.003	275	1 [Reference]	<.001	265		1 [Reference]		.12	.04	
Middle	318	−4.3 (−6.9 to −1.6)		154	−4.6 (−8.3 to −0.9)		164		−3.8 (−7.4 to −0.1)				
Low	328	−4.0 (−6.9 to −1.1)		172	−7.3 (−10.9 to −3.4)		156		−0.6 (−4.6 to 3.5)				
Paternal educational level^c^	1065			546			519						
High	401	1 [Reference]	.37	209	1 [Reference]	.046	192		1 [Reference]		.57	.07	
Middle	517	−1.0 (−3.6 to 1.6)		264	−0.5 (−4.0 to 3.1)		253		−1.5 (−5.0 to 2.3)				
Low	147	−2.8 (−6.6 to 1.1)		73	−6.3 (−11.1 to −1.1)		74		0.9 (−4.5 to 6.6)				
Neighborhood income^d^	1244	0.5 (−0.7 to 1.6)	.45	637	0.7 (−1.0 to 2.3)	.42	607		0.3 (−1.3 to 1.9)		.75	.71	

Abbreviations: SES, socioeconomic status; TL, telomere length.

^a^ Models adjusted for maternal age, paternal age, maternal prepregnancy body mass index, maternal smoking, parity, pregnancy complications, cesarean delivery, newborn sex, gestational age, birth weight, newborn race/ethnicity, and telomere batch.

^b^ Represents the overall *P* value for interaction for each newborn sex × SES indicator.

^c^ Estimates provided as a % difference (95% CI) in TL compared with the highest category (1 [Reference]).

^d^ Estimates provided as a % difference (95% CI) in TL for each SD increment in median annual household income (3374€).

In placental tissue, no individual SES measures were associated with TL in the total population (Table 4). However, in boys, maternal educational level (compared with high-level maternal educational level, difference in TL for middle-level maternal educational level, –3.8% [95% CI, –7.7% to 0.2%]; and difference in TL for low-level maternal educational level, –6.5% [95% CI, –12.0% to –0.7%]) and occupation (compared with high-level maternal occupation, difference in TL for middle-level maternal occupation, –2.0% [95% CI, –6.5% to 2.7%]; and difference in TL for low-level maternal occupation, –5.4% [95% CI, –9.9% to –0.7%]) were associated with placental TL, but no newborn sex interaction was observed. Socioeconomic status was not associated with placental TL in girls. Neighborhood income was not associated with cord blood and placental TL.

**Table 4.** Individual SES Indicators and Placental TL^a^

Indicator	No.	Total population		No.	Boys			No.		Girls			*P* value for interaction^b^	
		% Difference (95% CI)	*P* value		% Difference (95% CI)	*P* value			% Difference (95% CI)		*P* value		
Maternal educational level^c^	1258			646			612						
High	652	1 [Reference]	.22	317	1 [Reference]	.047	335		1 [Reference]		.90	.19	
Middle	467	−2.3 (−5.2 to 0.7)		246	−3.8 (−7.7 to 0.2)		221		−0.7 (−4.7 to 3.5)				
Low	139	−3.4 (−7.9 to 1.3)		83	−6.5 (−12.0 to −0.7)		56		0.8 (−6.0 to 8.1)				
Maternal occupation^c^	1186			601			585						
High	540	1 [Reference]	.23	275	1 [Reference]	.08	265		1 [Reference]		.35	.12	
Middle	318	0.6 (−2.7 to 4.1)		154	−2.0 (−6.5 to 2.7)		164		3.3 (−1.3 to 8.2)				
Low	328	−2.5 (−6.0 to 1.1)		172	−5.4 (−9.9 to −0.7)		156		0.5 (−4.4 to 5.5)				
Paternal educational level^c^	1065			546			519						
High	401	1	.38	209	1 [Reference]	.57	192		1 [Reference]		.56	.86	
Middle	517	0.4 (−2.8 to 3.6)		264	−0.5 (−4.7 to 4.0)		253		1.3 (−3.2 to 5.9)				
Low	147	−2.7 (−7.3 to 2.1)		73	−3.4 (−9.5 to 3.1)		74		−2.0 (−8.3 to 4.7)				
Neighborhood income^d^	1244	−0.7 (−2.1 to 0.7)	.31	637	−0.7 (−2.7 to 1.2)	.46	607		−0.7 (−2.6 to 1.2)		.45	.99	

Abbreviations: SES, socioeconomic status; TL, telomere length.

^a^ Models adjusted for maternal age, paternal age, maternal prepregnancy body mass index, maternal smoking, parity, pregnancy complications, cesarean delivery, newborn sex, gestational age, birth weight, newborn race/ethnicity, and telomere batch.

^b^ Represents the overall *P* value for interaction for each newborn sex × SES indicator.

^c^ Estimates provided as a % difference (95% CI) in TL compared with the highest category (1 [Reference]).

^d^ Estimates provided as a % difference (95% CI) in TL for each SD increment in median annual household income (3374€).

## Discussion

Our study highlights an important molecular mechanism that may explain the association between parental SES and lifelong health of the next generation. In the ENVIRONAGE birth cohort, we found that SES was associated with TL at birth. We observed a sex-specific association of SES and cord blood TL, with longer TL in boys in association with increased SES. For placental TL, we observed stronger associations in boys compared with girls, but no interaction for newborn sex was observed. Potential differences between cord and placental results may be owing to tissue-specific telomere-regulating differences during in utero development, including differences in telomerase activity, subtelomeric DNA methylation, or telomeric repeat-containing RNA expression.^28,29^ Our results suggest that prenatal SES is associated with TL in boys more explicitly than in girls, which may underlie a potentially higher susceptibility to disease later in life. A potential sex-related heterogeneity at birth may to some extent be in line with observations in adults supporting stronger SES associations in men compared with women, as most studies (18 of 20) indicate that male mortality is more unequal than female mortality across socioeconomic groups.^30^

Low maternal SES is associated with adverse pregnancy and neonatal outcomes,^31^ including higher risks for preterm births, low birth weight, small size for gestational age, respiratory distress, and increased morbidity and mortality rates, indicating the importance and consequences of a low SES from early life onward.^17,32,33,34^ In addition to these associations, we found that, on the level of telomere biology, SES is an important factor early in life. In adulthood, SES has been associated with TL; however, at birth results are inconsistent. Our results are in line with the observation that low maternal educational level was associated with shorter cord blood TL in a small population of 54 Latino infants.^19^ Our findings of a sex-specific association are supported by a recent report that observed an association between maternal educational attainment and cord blood TL in boys.^18^ In this latter study, annual household income was associated with cord blood TL in boys, which we could not confirm. Sex-specific differences in the association between SES and TL in adults are, however, inconclusive.^16,35,36^ Other studies evaluating cord blood TL^9^ or infant dried blood spot TL^20,21^ did not confirm our findings. However, these studies did not evaluate potential sex-specific associations. Currently, 1 study has reported on paternal educational level, and did not observe an association with cord blood TL.^37^ Although neighborhood disadvantages have been associated with shorter TL in adults,^38,39^ our data do not support an association between neighborhood income and TL at birth. This finding might be explained by the fact that individual SES parameters are of higher importance than surrounding SES measures.

Three lines of evidence show the importance of TL in early life. First, TL at birth is associated with later-life TL,^6^ indicating that TL-associated diseases may have their origins very early in life or even at birth. Second, in adults, baseline TL is associated with TL at later stages in life.^40^ Third, an animal-based study showed an association between early-life TL and lifespan, which may have considerable consequences if that were translatable to humans.^7^ Furthermore, large population-based studies suggest that a short TL later in life is associated with increased risks for cardiovascular disease and ^41^ type 2 diabetes^42^ and with increased mortality.^43^ Therefore, our results on SES and TL at birth may have important later-life health consequences.

How SES may be associated with TL is unclear. Telomeres are rich in guanine base pairs that are vulnerable to reactive oxygen species, leading to accelerated TL shortening.^44^ Low SES may reflect a number of exposures to chronic stress-inducing factors (including factors that lead to increased reactive oxygen species), whereas high SES is associated with exposures to more protective factors that may alter TL.^15^ Previous studies identified that maternal prepregnancy BMI^10^ and particulate air pollution exposure^11^ were associated with TL at birth. In other affluent societies, socioeconomic inequalities in BMI and other associated behavioral and environmental factors have been observed.^5,45^ In our study, mothers with low SES had a higher prepregnancy BMI. Formal mediation analysis showed that prepregnancy BMI partially mediated the association between SES and cord blood TL. A moderate mediation of prepregnancy BMI in cord blood but not placental tissue and the lack of explanation by smoking, air pollution, birth weight, and maternal diet and physical activity is in line with studies that observe weak or no mediating effects of these factors on the association of SES and adult or childhood TL.^35,46,47,48,49^ This finding may suggest that SES-related psychobiological chronic stress or epigenetic SES-related changes over generations are potentially involved in these associations.^50,51^ Although a meta-analysis showed that short-term perceived stress was weakly associated with TL, long-term chronic stress may have a large cumulative effect.^52^ Therefore, mothers with low SES may have an increased and faster accumulation of allostatic load and TL may represent a cellular memory capturing this cumulative history of oxidative stress and inflammation. These effects are potentially transferred from mother to newborn.^53^

Stronger associations between SES and TL in boys indicate potential stress-compensatory mechanisms or telomere maintenance mechanisms in girls that are absent in boys. Although girls and boys are susceptible to prenatal exposures, the ability to respond to and buffer against prenatal insults may be sex dependent.^54^ Higher levels of oxidative stress markers and a lower antioxidant capacity have been observed in newborn boys compared with girls.^55,56,57^ In addition, higher estrogen levels during the fetal development of girls may be important in regulating the protective capacity toward oxidative stress.^55,58^ Estrogens may scavenge free radicals, regulate antioxidant enzyme expression,^59^ and enhance telomerase activity,^60^ and are associated with longer infant TL.^61^

### Strengths and Limitations

This study has several strengths. We have a large (>1000) birth cohort with data on cord blood TL and placental TL. Our integrative SES measure retained its significance as a factor associated with newborn TL beyond numerous potential confounders and in several sensitivity analyses. Our results may be generalizable to affluent populations, as our population is representative for the population at large.^22^

This study also has some limitations. First, despite the large population in the study, increasing the population might reveal a more pronounced sex-specific different association for placental TL, which now was confirmed only for cord blood. Second, TL is heritable and parental TL is associated to some extent with newborn TL.^9^ As both maternal and paternal TL may be associated with their SES, this may mediate the observed association. This mediation could not be tested, as no data on parental TL are available. Third, we could not evaluate paternal occupational status, as no detailed job descriptions were available. Fourth, TL may be associated with childhood SES,^62,63^ and therefore parental childhood SES may be associated with our findings. Fifth, we can only speculate on the later-life consequences of TL at birth in association with health and disease, as currently long-term follow-up studies are lacking. Whether TL as measured in this study can causally be linked with later-life diseases is questionable and remains unproven; however, experimental studies show that dysfunctional telomeres can induce cardiomyopathy.^64^ Prospective follow-up studies are needed to evaluate whether TL at birth represents disease susceptibility later in life.^65,66^ Sixth, studying other biological markers of aging,^67^ including the epigenetic biomarker DNAm PhenoAge,^68^ could strengthen the evidence of the link between SES and aging. Seventh, other factors during pregnancy, such as hormones, oxidative stress, telomerase activity, and nutrition, warrant further evaluation in the interpretation of our results.

## Conclusions

Telomere length in early life sets later-life TL. Its potential importance for later-life disease susceptibility underscores the relevance of identifying early-life determinants of TL. Our results show a potential sex-specific association between parental SES and TL at birth, indicating a potential higher susceptibility in boys with a low SES at birth. Socioeconomic disadvantages in prenatal life may have potential lasting implications for molecular longevity or cellular aging.

## Notes

Supplement. **eMethods 1.** Description of Demographic and Perinatal Parameters **eMethods 2.** Detailed Coding of SES Measures **eMethods 3.** Cord Blood and Placental Sample Collection and Average Relative TL Measurement Using qPCR **eMethods 4.** Selection of Potential Mediators **eMethods 5.** Particulate Matter (PM_2.5_) Assessment **eTable.** Characteristics of the First Principal Component Reflecting the Integrative SES Variable **eFigure 1.** Flow Chart of Participant Selection **eFigure 2.** Heat Map of Correlations Between Different SES Indicators in the ENVIR*ON*AGE Birth Cohort (n = 1026) **eFigure 3.** Sensitivity Analysis for the Association Between Newborn TL and Integrative SES in the Entire Population **eFigure 4.** Sensitivity Analysis for the Association Between Newborn TL and Integrative SES in Newborn Boys and Girls **eReferences**
