# Supplementary material for: Association of Parental Socioeconomic Status and Newborn Telomere Length
Source: JAMA Netw Open. 2020 May 4;3(5):e204057. doi: 10.1001/jamanetworkopen.2020.4057 (PMC7199116; doi:10.1001/jamanetworkopen.2020.4057)
Supplement: Supplement. — eMethods 1. Description of Demographic and Perinatal Parameters eMethods 2. Detailed Coding of SES Measures eMethods 3. Cord Blood and Placental Sample Collection and Average Relative TL Measurement Using qPCR eMethods 4. Selection of Potential Mediators eMethods 5. Particulate Matter (PM2.5) Assessment eTable. Characteristics of the First Principal Component Reflecting the Integrative SES Variable eFigure 1. Flow Chart of Participant Selection eFigure 2. Heat Map of Correlations Between Different SES Indicators in the ENVIRONAGE Birth Cohort (n = 1026) eFigure 3. Sensitivity Analysis for the Association Between Newborn TL and Integrative SES in the Entire Population eFigure 4. Sensitivity Analysis for the Association Between Newborn TL and Integrative SES in Newborn Boys and Girls eReferences [file jamanetwopen-3-e204057-s001.pdf]

Supplementary Online Content

Martens DS, Janssen BG, Bijnens EM, et al. Association of parental socioeconomic status and newborn telomere length. *JAMA Netw Open.* 2020;3(5):e204057. doi:10.1001/jamanetworkopen.2020.4057

eMethods 1. Description of Demographic and Perinatal Parameters
eMethods 2. Detailed Coding of SES Measures
eMethods 3. Cord Blood and Placental Sample Collection and Average Relative TL Measurement Using qPCR
eMethods 4. Selection of Potential Mediators
eMethods 5. Particulate Matter ($PM_{2.5}$) Assessment
eTable. Characteristics of the First Principal Component Reflecting the Integrative SES Variable
eFigure 1. Flow Chart of Participant Selection
eFigure 2. Heat Map of Correlations Between Different SES Indicators in the ENVIR*ON*AGE Birth Cohort (n=1026)
eFigure 3. Sensitivity Analysis for the Association Between Newborn TL and Integrative SES in the Entire Population
eFigure 4. Sensitivity Analysis for the Association Between Newborn TL and Integrative SES in Newborn Boys and Girls
eReferences

This supplementary material has been provided by the authors to give readers additional information about their work.

© 2020 Martens DS et al. *JAMA Network Open.*

eMethods 1. Description of Demographic and Perinatal Parameters

Data on maternal pre-pregnancy weight, weight before delivery, height, pregnancy complication and cesarean sections were collected from the medical records at the hospital. Maternal height and weight were measured without shoes, wearing light clothes to the nearest centimeter and weight to the nearest 0.1 kg at the first antenatal visit of each pregnancy (weeks 7–9 of gestation). Maternal pre-pregnancy BMI was defined as weight in kilograms divided by the square of height in meters. Pregnancy complication was coded as “absent” when mothers did not experience any pregnancy complication or as “present” when mothers experienced one or more pregnancy complications. Included pregnancy complications were gestational diabetes, hypertension, infection diseases, pre-eclampsia, vaginal bleeding, and hyper- or hypothyroidism. Gestational age was estimated based on ultrasound data. Data was obtained on cesarean sections. Study questionnaires were completed to provide detailed information on maternal age, paternal age, maternal smoking status, parity, ethnicity, fruit and vegetables consumption and physical activity. Maternal consumption of fruit and vegetables was based on the number of portions as indicated by the mothers and classified into the following categories: less than 1 portion per day, 1 portion per day, 2 portions per day, and 3 or more portions per day. Maternal physical activity was based on the amount of times a week a mother indicated to be physically active for more than 20 minutes. This was coded as low, when mothers indicated to be active less than once a week, middle when indicated to be active once a week, and high when indicated to be active 2 or more times a week. Newborn ethnicity was based on the amount of European grandparents, ranging from 0 to 4. Perinatal parameters were obtained after birth such as birth date, newborn sex, birth weight.

© 2020 Martens DS et al. *JAMA Network Open.*

eMethods 2. Detailed Coding of SES Measures

We collected information about educational level and occupation by questionnaire. Maternal and paternal educational levels were coded as “low”, when parents only went to primary or secondary school without obtaining a degree, “middle” when they obtained a secondary school degree or “high” when they obtained a college or university degree. Maternal jobs were coded based on detailed job descriptions provided by the mothers. In case the mothers indicated not to have a job during pregnancy, we used the job description of the latest performed job by the mothers prior to pregnancy (on the average 9.4 months before pregnancy). We coded the following job rankings: “low”, including sales and customer service occupations, process, plant and machine operatives and elementary occupations, “middle”, including administrative and secretarial occupations, skilled trades occupations and caring, leisure and other service occupations, and “high”, including managers, directors, senior officials, professional occupations and associate professional and technical occupations. Based on the home address of the mothers, we assigned all mothers to statistical sectors (average area = 1.55 km^2), the smallest administrative entity for which statistical data are produced by the Belgian National Institute of Statistics. The province of Limburg, in which our recruitment is situated exists of 1396 statistical sectors of which our participants lived in 667 different statistical sectors, for which on the average 1034 declarations per sector were available. Belgian census-tract data (FOD Economie/DG Statistiek) derived from the NIS were used to define neighborhood income based on annual household income data (2014).

© 2020 Martens DS et al. *JAMA Network Open.*

eMethods 3. Cord Blood and Placental Sample Collection and Average Relative TL Measurement Using qPCR

Umbilical cord blood was drawn immediately after delivery in BD Vacutainer® plastic whole blood tubes with spray-coated K2EDTA (BD, Franklin Lakes, NJ, USA). Complete blood cell counts and differential leukocyte counts are determined using an automated cell counter with flow differential (Cell Dyn 3500, Abbott Diagnostics, Abott Park, IL, USA). Buffy coat was collected for DNA extraction after sample centrifugation at 3200 rpm for 15 min. Placental biopsies (1 to 2 cm^3) were taken directly underneath the chorioamniotic membrane for DNA extraction at the fetal side at approximately 4 cm from the umbilical cord. Care was taken by visual examination and dissection to avoid chorioamniotic membrane contamination. Histological examination of fetal placental biopsies confirmed that the placental sample tissue mainly contained cytothrophoblasts and syncytiotrophoblasts differentiated from trophoblasts. Placental and cord blood leukocyte DNA was extracted using the QIAamp DNA Mini Kit (Qiagen, Inc., Venlo, the Netherlands). DNA quantity and purity was assessed by a Nanodrop 1000 spectrophotometer (Isogen, Life Science, Belgium). DNA was considered pure when the A260/280 was greater than 1.80 and A260/230 greater than 2.0. DNA integrity was assessed by agarose gel-electrophoresis. To ensure a uniform DNA input of 5 ng for each qPCR reaction, samples were diluted and checked using the Quant-iT™ PicoGreen® dsDNA Assay Kit (Life Technologies, Europe). The telomere-specific qPCR reaction mixture contained 1x QuantiTect SYBR Green PCR master mix (Qiagen, Inc., Venlo, the Netherlands), 2 mM dithiothreitol (DTT), 300 nM telg primer (ACACTAAGGTTTGGGTTTGGGTTTGGGTTTGGGTTAGTG T) and 900 nM telc primer (TGTTAGGTATCCCTATCCCTATCCCTATCCCTATCCCTAACA). Cycling conditions used were: 1 cycle at 95°C for 10 min, followed by 2 cycles at 94°C for 15 sec and 49°C for 2 min and 30 cycles at 94°C for 15 sec, 62°C for 20 sec, and 74°C for 1 min and 40 sec. The single-copy gene qPCR mixture contained 1x QuantiTect SYBR Green PCR master mix, 300 nM 36B4u primer (CAGCAAGTGGGAAGGTGTAATCC) and 500 nM 36B4d primer (CCCATTCTATCATCAACGGGTACAA) . Used cycling conditions were: 1 cycle at 95°C for 10 min, followed by 40 cycles at 95°C for 15 sec, and 58°C for 1 min and 20 sec. All measurements were performed in triplicate on a 7900HT Fast Real-Time PCR System (Applied Biosystems) in a 384-well format. After each qPCR a melting curve analysis was performed. On each run, a 6-point serial dilution of pooled buffy coat or pooled placental DNA was run to assess PCR efficiency as well as eight inter-run calibrators to account for inter-run variability. qPCR curves for each sample were visually inspected and when technical problems were detected or triplicates showed too high variability, samples were removed for further analysis (n=18 for cord blood and n=28 for placental TL). Telomeres were measured in two separate batches, and were calculated using qBase (Biogazelle, Zwijnaarde, Belgium). The reliability of our assay was assessed by calculating the interclass coefficient (ICC) with 95% CI of triplicate measures (T/S ratios, T and S measures separately) for both cord blood and placental telomeres using the SPSS statistical package version 25 (IBM Corp. Armonk, NY, USA) based on a mean rating, absolute-agreement, 2-way mixed-effects model. The ICC (95% CI) of cord blood T/S ratios, telomere runs and single-copy runs were 0.954 (0.949 to 0.959), 0.989 (0.988 to 0.990) and 0.984 (0.981 to 0.986), respectively. The ICC (95% CI) of placental T/S ratios, telomere runs, single-copy gene runs were 0.96 (0.955 to 0.964), 0.986 (0.984 to 0.987) and 0.98 (0.976 to 0.983), respectively. Based on the eight inter-run calibrators, the inter-assay ICC was 0.941 (0.853 to 0.988) for cord blood telomeres and 0.979 (0.953 to 0.995) for placental telomeres.

© 2020 Martens DS et al. *JAMA Network Open.*

eMethods 4. Selection of Potential Mediators

We hypothesized that maternal pre-pregnancy BMI, maternal smoking and birth weight are potential mediators that may underlie the relationship between the integrative SES (exposure) and newborn TL (outcome). Furthermore, we considered other variables available in the ENVIR*ON*AGE birth cohort to be potential mediators. These included maternal residential air pollution exposure (PM2.5) (see eMethods 5, available for n=891), and variables reflecting physical activity (n=990) and maternal diet (n=992). The latter two were based on self-reported activity and fruit and vegetables consumption (see eMethods 1 for definition). We previously showed the link between air pollution exposure and maternal pre-pregnancy BMI and newborn TL in the ENVIR*ON*AGE birth cohort.[1,2]

© 2020 Martens DS et al. *JAMA Network Open.*

eMethods 5. Particulate Matter ($PM_{2.5}$) Assessment

Based on the mother's residential address, daily mean $PM_{2.5}$ concentrations (in micrograms per cubic meter) were estimated using a high-resolution spatial-temporal interpolation method (kriging)[3] in combination with a dispersion model.[4,5] This interpolation method uses hourly measured $PM_{2.5}$ pollution data collected at the official fixed-site monitoring stations (n=34) and land-cover data obtained from satellite images.[6] The model chain provides daily $PM_{2.5}$ values on a dense, irregular receptor grid by using data both from the Belgian telemetric air-quality network and emissions from point sources and line sources. In the Flemish region of Belgium, more than 80% (R^2 = 0.8) of the temporal and spatial variability was explained by this interpolation tool.[7] From the date of conception onward, a mean $PM_{2.5}$ concentration for the entire duration of pregnancy was calculated using daily mean $PM_{2.5}$ concentrations at the mother's residence.

© 2020 Martens DS et al. *JAMA Network Open.*

eTable. Characteristics of the First Principal Component Reflecting the Integrative SES Variable

	**Eigenvalues**	**Variance explained**	**Individual SES measures**	**Loadings**
PC1, integrative SES	2.20	59%	Maternal education	0.85
			Maternal occupation	0.85
			Paternal education	0.71
			Neighborhood income	0.48

© 2020 Martens DS et al. *JAMA Network Open.*

eFigure 1. Flow Chart of Participant Selection

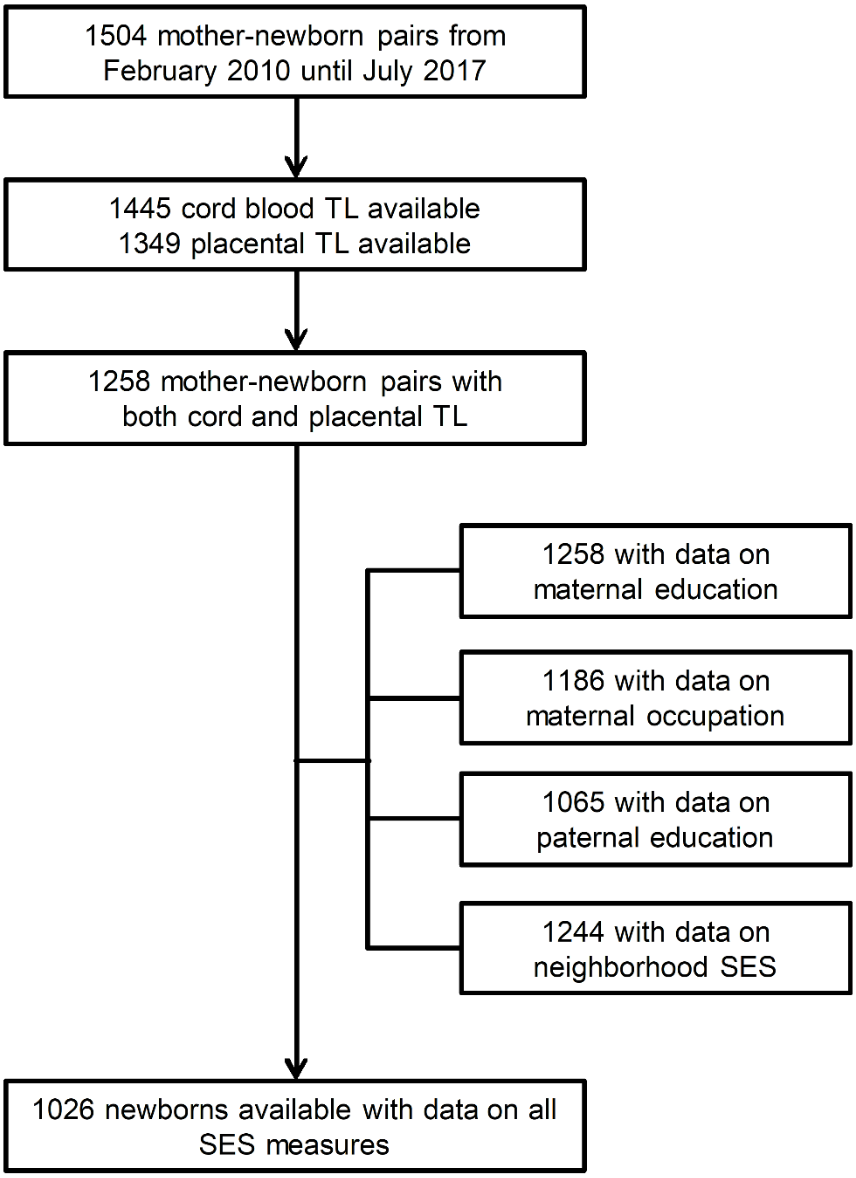

In total 1463 cord blood and 1377 placental samples were collected, but n=18 cord blood and n=28 placental TLs were excluded due to too high variability. Data was missing on 48 maternal jobs, and 24 students were not categorized into maternal occupation. Education was missing for 193 fathers.

© 2020 Martens DS et al. *JAMA Network Open.*

eFigure 2. Heat Map of Correlations Between Different SES Indicators in the ENVIR*ON*AGE Birth Cohort (n=1026)

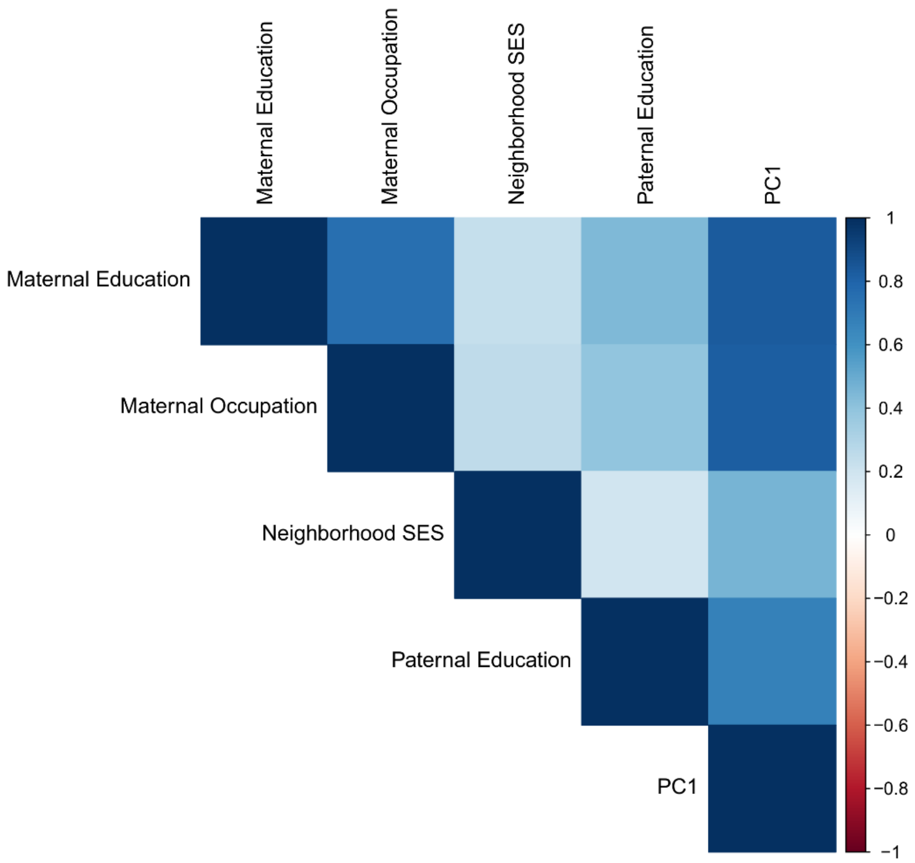

Each colored square represents the correlation coefficient between different SES indicators.

© 2020 Martens DS et al. *JAMA Network Open.*

eFigure 3. Sensitivity Analysis for the Association Between Newborn TL and Integrative SES in the Entire Population

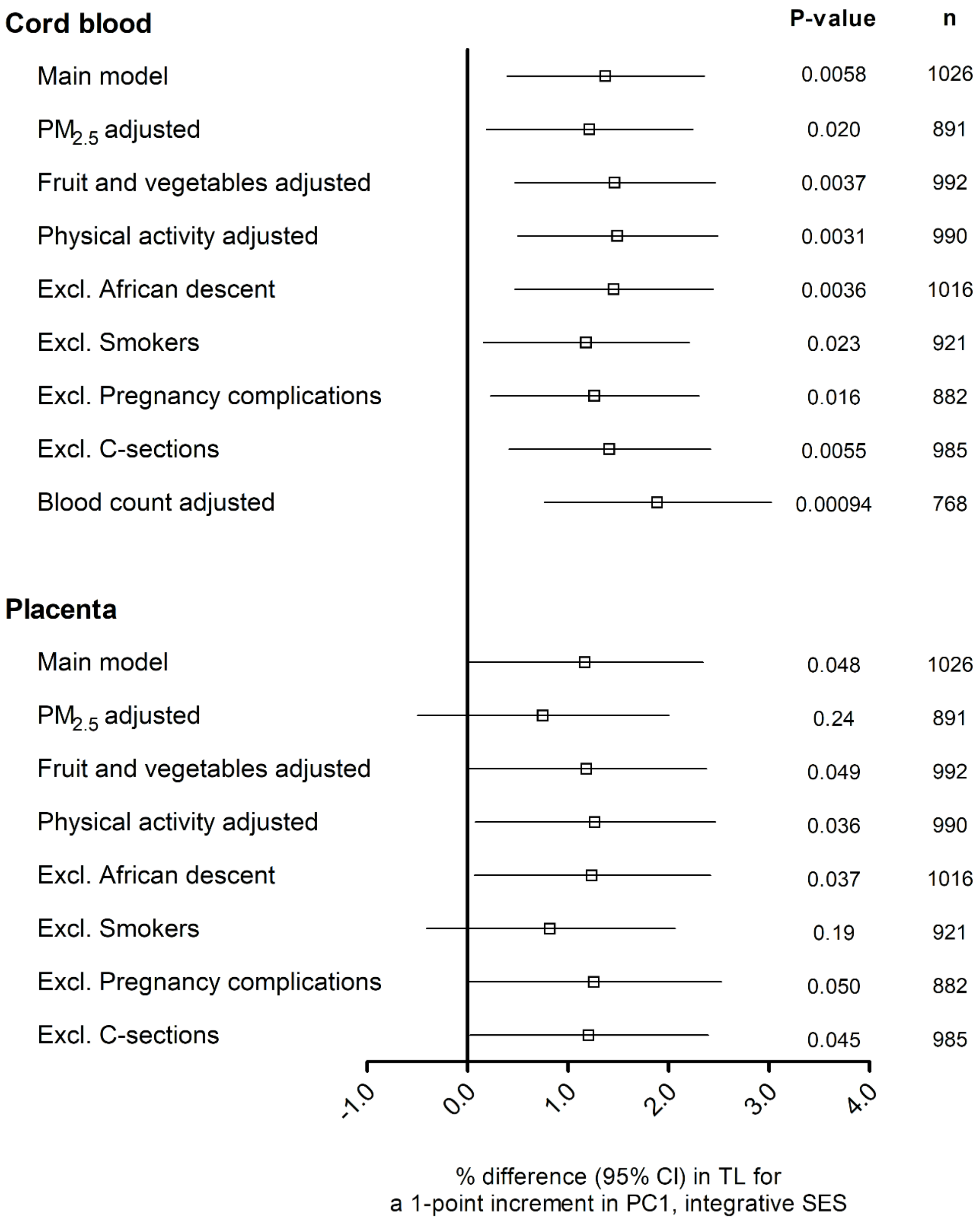

Models adjusted for parental age, maternal pre-pregnancy BMI, maternal smoking, parity, pregnancy complications, cesarean section, newborn sex, gestational age, birth weight, newborn ethnicity, and batch. Models adjusted for blood count included white blood cells (log10 count), neutrophils (%), lymphocytes (%), monocytes (%) and eosinophils (%).

© 2020 Martens DS et al. *JAMA Network Open.*

eFigure 4. Sensitivity Analysis for the Association Between Newborn TL and Integrative SES in Newborn Boys and Girls

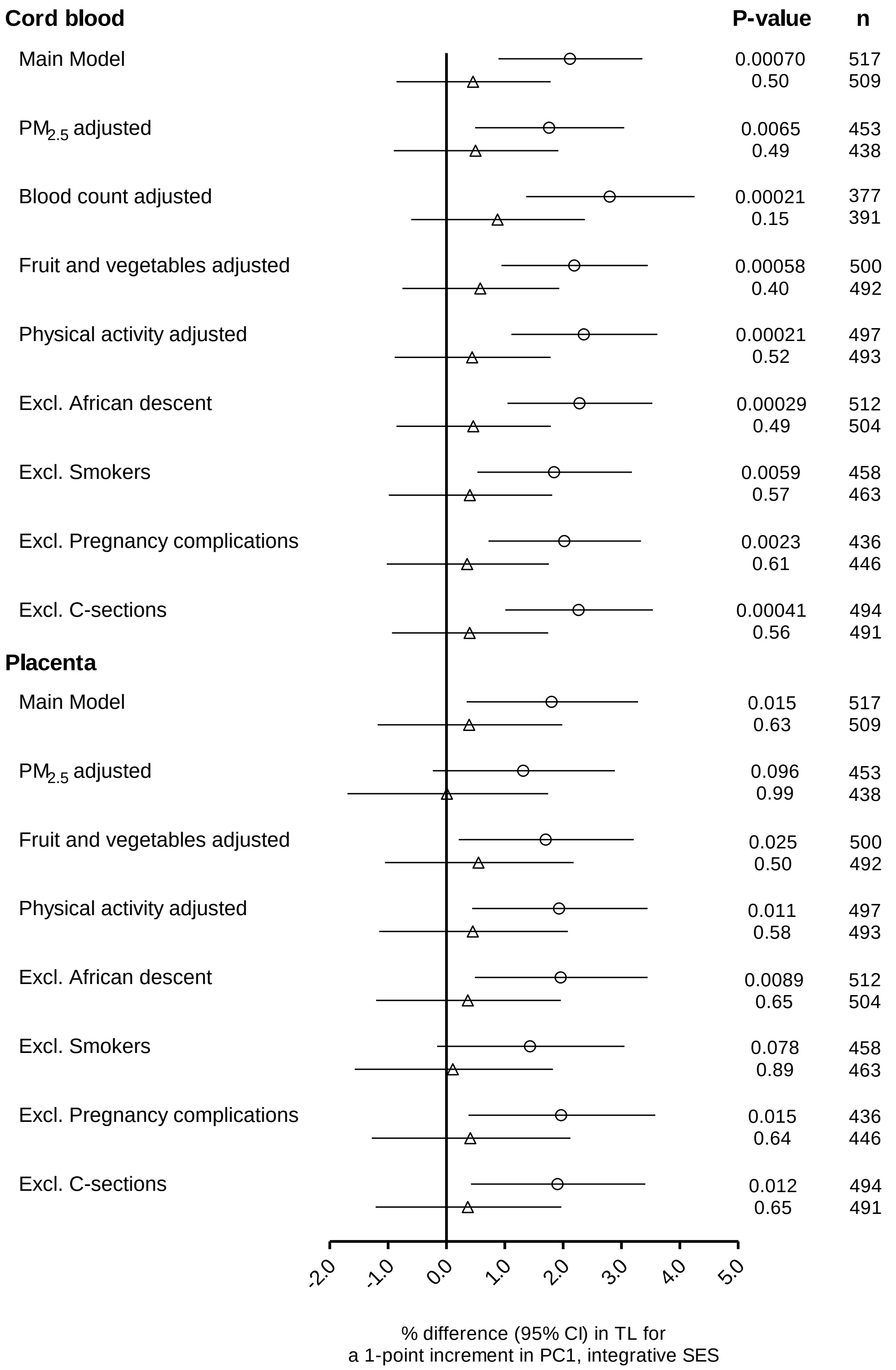

Estimates presented as a % difference (with 95% CI) in TL for a 1-point increment in integrative SES for newborn boys (o) and newborn girls (Δ). Estimates retrieved from the newborn sex*integrative SES interaction model. Models presented were fully adjusted for maternal age, paternal age, maternal pre-pregnancy BMI, maternal smoking, parity, pregnancy complications, cesarean section, newborn sex, gestational age, birth weight, newborn ethnicity and batch. Models adjusted for blood count included white blood cells (log_{10} count), neutrophils (%), lymphocytes (%), monocytes (%) and eosinophils (%).

© 2020 Martens DS et al. *JAMA Network Open.*

eReferences

1. Martens DS, Cox B, Janssen BG, et al. Prenatal Air Pollution and Newborns' Predisposition to Accelerated Biological Aging. *JAMA Pediatr.* 2017;171(12):1160-1167.
2. Martens DS, Plusquin M, Gyselaers W, De Vivo I, Nawrot TS. Maternal pre-pregnancy body mass index and newborn telomere length. *BMC Med.* 2016;14(1):148.
3. Janssen S, Dumont G, Fierens F, Mensink C. Spatial interpolation of air pollution measurements using CORINE land cover data. *Atmospheric Environment.* 2008;42(20):4884-4903.
4. Lefebvre W, Degrawe B, Beckx C, et al. Presentation and evaluation of an integrated model chain to respond to traffic- and health-related policy questions. *Environmental Modelling & Software.* 2013;40(0):160-170.
5. Lefebvre W, Vercauteren J, Schrooten L, et al. Validation of the MIMOSA-AURORA-IFDM model chain for policy support: Modeling concentrations of elemental carbon in Flanders. *Atmospheric Environment.* 2011;45(37):6705-6713.
6. European Environmental Agency. Corine land cover. https://www.eea.europa.eu/publications/COR0-landcover.Accessed January 30, 2018.
7. Maiheu BV, B. Viane, P. De Ridder, K. Lauwaet, D. Smeets, N. Deutsch, F. Janssen, S. Identifying the best available large-scale concentration maps for air quality in Belgium [in Dutch]. *http://wwwmilieurapportbe/Upload/main/0_onderzoeksrapporten/2013/Eindrapport_Concentratiekaarten_29_01_2013_TWpdf.*Published December 2012:Accessed January 30, 2018.

© 2020 Martens DS et al. *JAMA Network Open.*
